# Correlation of cavotricuspid isthmus dynamics with clinical parameters: insights from interventional cardiac magnetic resonance imaging

**DOI:** 10.1093/ehjimp/qyaf106

**Published:** 2025-10-08

**Authors:** Andrei Alexandru Mircea, Agathe Pusturle de Cidrac, Adrian Luca, Cheryl Teres, Patrizio Pascale, Mathieu Le Bloa, Ciro Ascione, Giulia Domenichini, Mattia Pagnoni, Alessandra Pia Porretta, Cosima Jahnke, Kerstin Bode, Sabrina Oebel, Frank Lindemann, Panagiotis Antiochos, Ambra Masi, Jorge Solana-Muñoz, Juerg Schwitter, Etienne Pruvot, Ingo Paetsch

**Affiliations:** Service of Cardiology, Lausanne University Hospital and University of Lausanne, Rue du Bugnon 46, Lausanne 1011, Switzerland; Institut Hospitalo-Universitaire Liryc, Xavier Arnozan Hospital, Haut Lévêque Avenue, Pessac 33604, France; Service of Cardiology, Lausanne University Hospital and University of Lausanne, Rue du Bugnon 46, Lausanne 1011, Switzerland; Bioengineering and Biomedical Engineering Department, Ecole Polytechnique Fédérale de Lausanne, Lausanne 1015, Switzerland; Service of Cardiology, Lausanne University Hospital and University of Lausanne, Rue du Bugnon 46, Lausanne 1011, Switzerland; Service of Cardiology, Lausanne University Hospital and University of Lausanne, Rue du Bugnon 46, Lausanne 1011, Switzerland; Service of Cardiology, Lausanne University Hospital and University of Lausanne, Rue du Bugnon 46, Lausanne 1011, Switzerland; Faculty of Biology and Medicine, University of Lausanne, Lausanne 1015, Switzerland; Service of Cardiology, Lausanne University Hospital and University of Lausanne, Rue du Bugnon 46, Lausanne 1011, Switzerland; Service of Cardiology, Lausanne University Hospital and University of Lausanne, Rue du Bugnon 46, Lausanne 1011, Switzerland; Service of Cardiology, Lausanne University Hospital and University of Lausanne, Rue du Bugnon 46, Lausanne 1011, Switzerland; Service of Cardiology, Lausanne University Hospital and University of Lausanne, Rue du Bugnon 46, Lausanne 1011, Switzerland; Service of Cardiology, Lausanne University Hospital and University of Lausanne, Rue du Bugnon 46, Lausanne 1011, Switzerland; CNMR Maladies Cardiaques Héréditaires Rares, APHP, Hôpital Bichat Claude-Bernard, Paris, France; Heart Center, University Hospital Leipzig, Leipzig 04289, Germany; Heart Center, University Hospital Leipzig, Leipzig 04289, Germany; Heart Center, University Hospital Leipzig, Leipzig 04289, Germany; Heart Center, University Hospital Leipzig, Leipzig 04289, Germany; Service of Cardiology, Lausanne University Hospital and University of Lausanne, Rue du Bugnon 46, Lausanne 1011, Switzerland; Cardiac MR Center of the University Hospital Lausanne, CRMC, Lausanne, Switzerland; Service of Cardiology, Lausanne University Hospital and University of Lausanne, Rue du Bugnon 46, Lausanne 1011, Switzerland; Cardiac MR Center of the University Hospital Lausanne, CRMC, Lausanne, Switzerland; Service of Cardiology, Lausanne University Hospital and University of Lausanne, Rue du Bugnon 46, Lausanne 1011, Switzerland; Service of Cardiology, Lausanne University Hospital and University of Lausanne, Rue du Bugnon 46, Lausanne 1011, Switzerland; Faculty of Biology and Medicine, University of Lausanne, Lausanne 1015, Switzerland; Cardiac MR Center of the University Hospital Lausanne, CRMC, Lausanne, Switzerland; Service of Cardiology, Lausanne University Hospital and University of Lausanne, Rue du Bugnon 46, Lausanne 1011, Switzerland; Faculty of Biology and Medicine, University of Lausanne, Lausanne 1015, Switzerland; Heart Center, University Hospital Leipzig, Leipzig 04289, Germany

**Keywords:** interventional MRI, atrial flutter, cavotricuspid isthmus, K-means clustering, normalized elongation, electrical cardioversion

## Abstract

**Aims:**

Ablation of typical atrial flutter (AFL) within an interventional cardiac magnetic resonance (iCMR) is a novel treatment modality. This study aims to describe the segmental kinetics of the cavotricuspid isthmus (CTI) during iCMR-guided ablation for AFL, to evaluate the impact of CTI dynamics on procedural time, to assess the utility of machine learning (ML) for clustering patient profiles based on CTI kinetics, and to identify clinical factors influencing CTI kinetics.

**Methods and results:**

A cohort of 32 patients underwent first-time iCMR-guided CTI ablation while in sinus rhythm, of whom 15 (47%) underwent a successful electrical cardioversion (EC) within 12 h before the procedure. CTI delineation and measurements were retrospectively performed using TOMTEC-ARENA™ software. Normalized elongation (NE) was defined as the ratio between CTI elongation and CTI length during right atrial systole. Unsupervised ML (K-means clustering) was used for patients’ classification.

Segmental analysis revealed greater displacements for CTI segments near the tricuspid valve compared with those near the Eustachian valve. K-means clustering identified three patient groups: low, intermediate, and high NE. Prior EC was significantly associated with low NE (*P* < 0.05), suggesting myocardial stunning. Hypokinetic CTIs were more prevalent among patients with dyslipidaemia, smoking history, and elevated BMI.

**Conclusion:**

This study provides the first detailed description of segmental CTI dynamics during iCMR-guided AFL ablation. NE emerged as a valuable metric for characterizing CTI kinetics. A clinical profile including a history of EC, smoking status, elevated BMI, and dyslipidaemia, was linked to reduced CTI kinetics suggestive of right atrial cardiomyopathy.

## Graphical Abstract

**Graphical Abstract:**
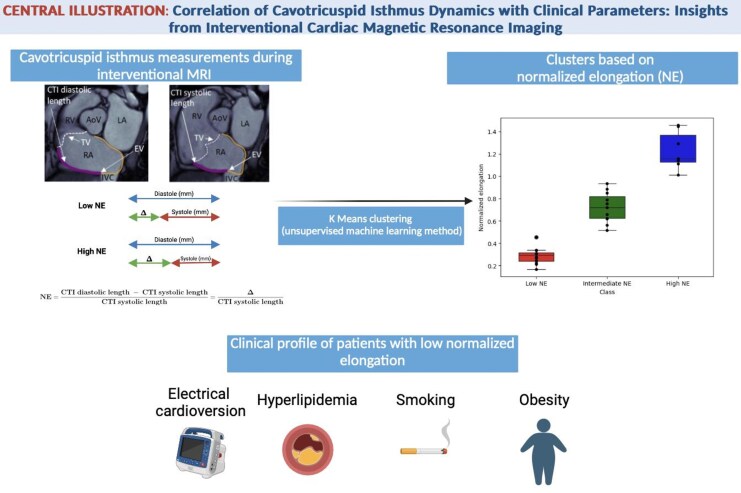

## Introduction

Cardiac magnetic resonance imaging (CMR) provides accurate anatomic details for the characterization of cardiac arrhythmogenic substrate and radiofrequency (RF) lesions.^1,2^ Cavotricuspid isthmus (CTI) ablation for typical atrial flutter (AFL) was the first implementation of ablative methods within an interventional CMR (iCMR),^3^ which involves the use of CMR-compatible devices and trained staff.^4^ CTI kinetic properties remain poorly known in normal subjects as well as in patients with AFL. First, whether CTIs display homogeneous kinetics has not been studied yet. Secondly, there are no known CTI kinetic parameters for characterizing tissue compliance, for describing patients with AFL and for predicting the difficulty of CTI ablation. Thirdly, whether comorbidities or electrical cardioversion (EC) impact CTI contractility is not yet known. This study aims to describe the segmental kinetics of CTI during iCMR-guided ablation for AFL, to evaluate the impact of CTI kinetics on procedural time, to assess the utility of machine learning (ML) for clustering patients’ profiles based on CTI kinetics, and to identify clinical factors influencing CTI kinetics.

## Methods

### Study population, RF ablation, and success criteria

This single-centre, retrospective, observational study included 32 patients (66 ± 10 years, 29 males) with isthmus-dependent AFL referred for first-time CTI ablation. Exclusion criteria were: (i) any general contraindications for CMR imaging (i.e. implanted pacemaker/cardioverter defibrillator), and (ii) a stroke within the last 6 months. All patients underwent an iCMR guided ablation procedure at the Leipzig Heart Center, Germany, between September 2021 and May 2023, which is outlined in Paetsch *et al*.^3^ and in Supplementary Methods. The study was approved by the local ethical committee (reference 109/20-ek). The procedural endpoint was the achievement of a bidirectional CTI block verified by pacing maneuvers and post-ablation mapping within the iCMR. Clinical success was defined as the absence of recurrence of typical isthmus-dependent AFL at 3-month post-procedure follow-up. Procedures were performed by seven electrophysiologists with an average of 10 years of experience. Of the 32 patients, 15 (47%) underwent EC within 12 h before ablation to restore sinus rhythm.^5^ Hence, all patients were in sinus rhythm at the time of the EP-CMR-guided RF ablation (RFA).

### CTI metrics

CMR images were acquired and registered as standard DICOM files. A 2D cine loop, consisting of 25 timestamps from the entire cardiac cycle, was acquired using a tailored geometry derived from a short-axis view of the heart. This special axis-view provides visualization of several key cardiac structures including the right atrium (RA), the CTI, the right ventricle (RV), the tricuspid (TV), and the Eustachian (EV) valve, the inferior vena cava (IVC), the left atrium (LA), and the aortic valve as shown in Supplementary data online, *Figure S1*. Motion analysis and data acquisition were performed with TOMTEC-ARENA™ software. Complete delineation of the RA wall began by manually placing points along its contour. Delineation of the RA wall was done on the images corresponding to the systolic and diastolic temporal frames. The software automated the contouring process of the RA wall across the remaining 23 images. TOMTEC automatically placed 49 points along the RA contour (see Supplementary data online, *Figure S2*). This odd number arises from the inclusion of a critical point located near the IVC, along with 24 additional points on each side. X and Y co-ordinates of the 49 points over the 25 images were generated by the software. Data output contained information for each of the 49 points along the RA wall as well as for the 25 temporal images, including position and instantaneous velocity in an orthogonal X and Y co-ordinate system, and displacement and velocity in transverse and longitudinal directions. All CTI measurements were performed by two cardiologists (P.A. and A.M.) with >5 years of CMR experience.

**Figure 1:**
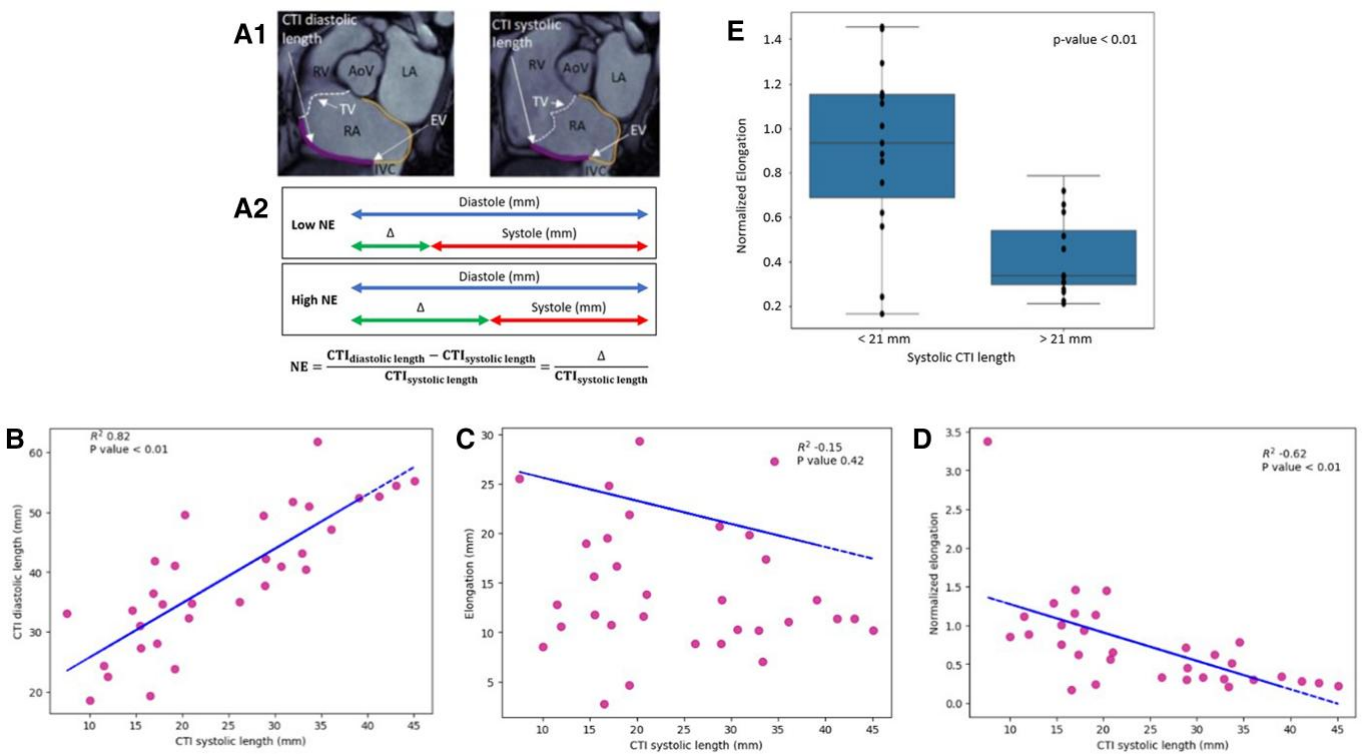
CTI metrics. (*A*) CTI measurement methodology (*A*1) Method for MRI measurements in diastole and systole (*A*2) NE. (*B*) Relationship between CTI diastolic and systolic length. (*C*) Relationship between elongation and CTI systolic length. (*D*) Relationship between NE and CTI systolic length. (*E*) NE based on CTI systolic length.

**Figure 2:**
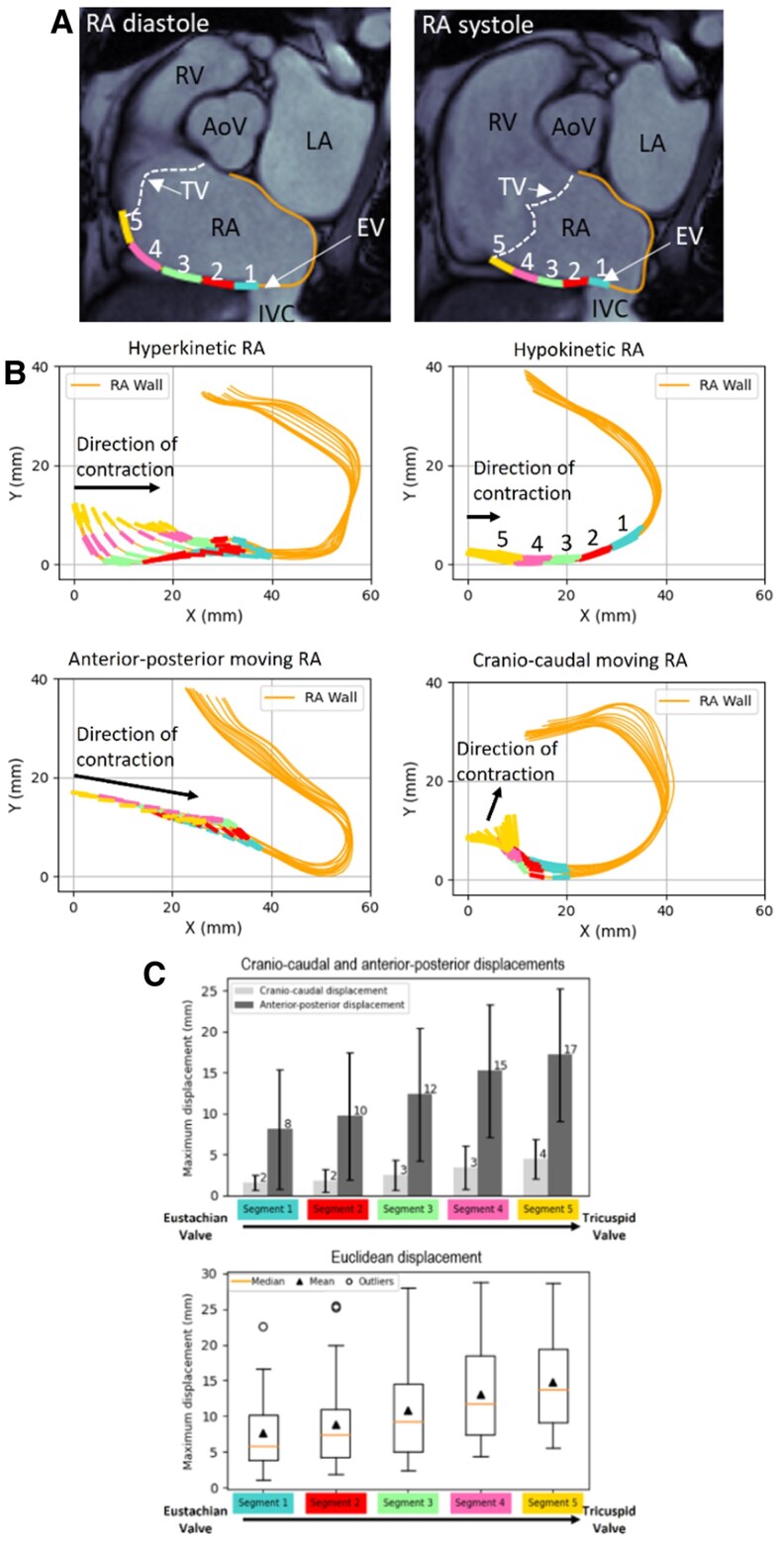
CTI segmental dynamics. (*A*) CMR short axis views of the RA wall and CTI divided in five segments during RA diastolic (left) and systolic (right) phases (CTI, cavotricuspid isthmus; RV, right ventricle; TV, tricuspid valve; RA, right atrium; IVC, inferior vena cava; LA, left atrium; AoV, aortic valve; EV, Eustachian valve). (*B*) Representative examples of RA motion patterns: hyperkinetic RA (upper left), hypokinetic RA (upper right), predominant antero-posterior motion (lower left), and predominant cranio-caudal motion (lower right). (*C*) Upper panel: average CTI maximum displacements in caudo-cranial and postero-anterior directions for the study population. Lower panel: maximum CTI displacements for the study population.

CTI length was measured in RA systole and diastole using two different methods: (i) the ‘curvilinear’ CTI length measured as the cumulated distance between consecutive points of the CTI, and (ii) ‘linear’ CTI length defined as the Euclidean distance between the IVC and the TV hinge points (*Figure 1A1*). The depth of the CTI pouch was measured as the maximum distance between CTI points and the straight line used to define the ‘linear’ CTI length. Of note, only the linear CTI length and the pouch depth measurements were found in the literature. These measures were meant to confirm that our cohort was within the ranges previously seen. We are using the curvilinear length for the descriptive analysis of the CTI kinetics. These measures better represent the CTI shape and enable a direct calculation of CTI elongation and NE. The predictive models will make use of all measures. CTI elongation was calculated as the difference between the curvilinear CTI length in RA diastole and that in RA systole. Normalized elongation (NE) was defined as the ratio between CTI elongation and CTI length during RA systole as shown in *Figure 1A2*. NE serves as a biomechanical marker of tissue compliance. Specifically, it is used within the context of linear stress-strain. CTI kinetics were analysed by dividing each CTI into five segments (*Figure 2A* and Supplementary data online, *Figure S3*). Comparison between CTI segments was performed to highlight specific motion patterns such as the extent of contraction and relaxation. CTI displacement over the cardiac cycle was assessed by computing the difference between the final and the initial position of each CTI point. CTI segmental displacement was then calculated by averaging the values of all points within each segment. The trajectory differential was defined as the change in position of a CTI point between two consecutive time frames of the cardiac cycle. For instance, point 1 (P1) from a specific CTI segment has trajectory differentials between time *n* and time *n-1* (for *n* = 1, …, 25) such as:


$$\mathrm{Trajectory}\;\;\mathrm{Differential}\;\;(\mathrm{of}\;\;P1\;\;\mathrm{on}\;\;t=n)=\Delta_{n(P1)}=\mathrm{Position}(t_{n})-\;\;\mathrm{Position}(t_{n-1})$$

As the CMR slices are bi-dimensional, the trajectory differentials have two axes.

The instantaneous velocity of each CTI point was defined as its velocity at a specific time *n* (for *n* = 1, …, 25).

### Relationship of CTI metrics with procedural and clinical parameters

To test the relationship between NE and various procedural and clinical parameters, several statistical methods were applied. First, the Spearman correlation test was used to evaluate the relationship between procedural time and NE or absolute elongation. Procedural time (in minutes) was defined as the period between insertion of the first catheter to removal of the last catheter from the patient. Secondly, the point bi-serial function was used to assess the relationship between: (i) NE and dichotomous/categorical variables such as EC, smoker status, dyslipidaemia, NYHA, and BMI class; and (ii) absolute CTI elongation and NYHA class. Dyslipidaemia was defined as a treated disorder or as a treatment indication according to current guidelines.^6^ Plots were used to visualize the impact of EC status on the velocity and trajectory differential on both axes.

### Understanding the impact of clinical and procedural parameters on predicting CTI kinetics and metrics

Logistic regression was the most appropriate model to use for the categorical prediction of whether the patient was cardioverted or not. The input was the vector of trajectory differentials for each one of the CTI segmented points. A prediction algorithm was developed using the Lasso regression model to predict either the CTI diastolic or systolic length.^7^ The Lasso model was chosen as it provides easily interpretable coefficients, highlighting whether the procedural and clinical features provide additive value. The model used an optimal alpha determined through 10-fold cross-validation. The alpha values were 0.65 for CTI diastolic length prediction and 0.17 for CTI systolic length prediction. The inputs for predicting CTI systolic length were (i) curvilinear CTI diastolic length, (ii) CTI pouch depth, (iii) linear CTI length, (iv) EC in the last 12 h, (v) smoker status, and (vi) dyslipidaemia status. The inputs for predicting CTI diastolic length were the same with the exception of using CTI systolic length. The model was trained with and without clinical and procedural inputs as well.

### Statistical analysis and ML methods

Continuous variables were expressed as mean and standard deviation or as median with inter-quartile ranges (IQR), while categorical variables were reported as numbers and percentages. Correlations between continuous variables were assessed using the Spearman rank test, and relationships between continuous and dichotomous/categorical variables were evaluated with the point-biserial test. Data non-uniformity was tested using the Kolmogorov–Smirnov test. The statistical analysis and ML methods were performed in Python. Among the various Python libraries employed in this study, two are particularly noteworthy. The ‘scikit-learn’^8^ facilitated both supervised and unsupervised ML tasks. The ‘scipy.stats’^9^ was utilized for statistical analyses. The K-means clustering algorithm was used to classify patients based on their CTI properties. This unsupervised ML method optimizes the classification by minimizing the distance between observations and their respective centroids, while maximizing the distance between centroids (i.e. clusters). This process is illustrated in Supplementary data online, *Figure S4*, which shows the within-cluster sum of squares, computed as the Euclidean distance between centroids and its associated observations. The elbow method consists of identifying the inflection point corresponding to the optimal cluster groups. In this case, four distinct cluster groups were identified based on NE as the discriminant criterion (see Supplementary data online, *Figure S4*).

## Results

### Clinical characteristics and ablation data

The clinical characteristics and the ablation data of the study population are summarized in Table 1. Of the 32 study patients, 17 had dyslipidaemia and 17 were smokers. The average body mass index (BMI) was 29 ± 4. Procedural data are detailed in Table 2, with no complications reported during or after the intervention.

**Table 1:** Clinical characteristics of the study population

Age (years)	66 ± 10
Male gender	29 (90%)
BMI (kg/m^2^)	29 ± 4
Left ventricular ejection fraction (%)	61 ± 5
Hypertension	29 (91%)
Diabetes	9 (28%)
Coronary artery disease	7 (22%)
Smokers	17 (53%)
Dyslipidaemia	17 (53%)
Hemodynamic data (onset of procedure)	
Heart rate (bpm)	68 ± 7
Systolic blood pressure (mmHg)	123.3 ± 21.5
Diastolic blood pressure (mmHg)	63.4 ± 9.9

**Table 2:** RFA procedural characteristics

EC before ablation, *n* (%)	15 (46.87)
Bidirectional isthmus block, *n* (%)	32 (100)
Mean procedural ablation duration (min)	18.9 ± 10.5
Mean procedural time (min)	44.8 ± 22.5
Number of operators	7

### CTI anatomical metrics, prior interventions, and their relationship with procedural time

Table 3 presents the anatomical CTI characteristics. The mean and median curvilinear systolic CTI lengths (see Supplementary materials; *Figure 1B*) were 24.6 ± 10.4 and 21 mm (IQR = 16.21), respectively. The mean and median diastolic CTI lengths were 40.0 ± 11.4 and 39 mm (IQR = 17.49), respectively. In comparison, the mean and median linear CTI diastolic lengths were 36.2 ± 9.8 and 36 mm (IQR = 11.42), respectively. *Figure 1B* depicts the relationship between CTI length in systole and diastole, showing a strong positive correlation (*R*^2^ = 0.82, *P* < 0.01). The longer the CTI length in diastole, the greater its length in systole.

**Table 3:** CTI anatomical characteristics of the study population

		Mean ± SD	Median (IQR)
Curvilinear CTI length (mm)	RA systole	24.6 ± 10.4	21.0 (16.2)
	RA diastole	40.0 ± 11.4	39.0 (17.5)
Linear CTI length (mm)	RA diastole	36.2 ± 9.8	36.0 (11.4)
CTI pouch depth (in RA diastole) (mm)		3.2 ± 2.0	2.7 (2.6)
Elongation (mm)		14.4 ± 6.5	12.3 (8.8)
NE		0.7 ± 0.6	0.6 (0.6)

Hereafter, CTI length refers exclusively to the measures performed with the ‘curvilinear’ method. The NE had a mean value of 0.74 ± 0.61 and a median one of 0.6 (Table 3). There was no correlation between absolute CTI elongation and CTI systolic length (*Figure 1C*, *R*^2^ = −0.15, *P* = 0.42). Interestingly, some short and long systolic CTIs exhibited similar elongation values. Conversely, a negative linear relationship was observed between systolic CTI length and NE values (*Figure 1D*, *R*^2^ = −0.62, *P* < 0.01), which shows that longer systolic CTI lengths are associated with lower NE values.

The study population was divided into two groups based on the median systolic CTI length value (21 mm). Patients with a systolic CTI length <21 mm had a significantly higher NE than those with a systolic CTI length >21 mm (0.91 ± 0.38 vs. 0.42 ± 0.18, *P* < 0.05) as shown in *Figure 1E*. One patient was excluded from this analysis because of his outlier NE value (3.38).

The mean total ablation duration was 18.9 ± 10.5 min, while the mean procedural time was 44.8 ± 22.5 min. Supplementary data online, *Figure S5* illustrates the lack of significant correlation between mean procedural time and both CTI NE and absolute elongation. Conversely, prior EC and procedural time showed a trend for an inverse correlation (correlation coefficient of −0.32, *P* = 0.08), suggesting shorter ablation procedures for EC patients. Supplementary data online, *Figure S6* highlights the relationships between NE, EC, and procedural time.

### Segmental CTI analysis

A key observation concerned the variation in CTI kinetics among patients as shown in Supplementary data online, *Figure S3*. *Figure 2B* categorizes patients based on distinct kinetics patterns, including hypo- and hyperkinetic CTI, as well as predominant anterior-posterior and caudo-cranial CTI displacements. These motion patterns can be categorized as follows:

1. Hyperkinetic CTI: exhibited pronounced displacement, indicating vigorous wall motion kinetics.
2. Hypokinetic CTI: displayed reduced displacement amplitude, reflecting weak wall motion kinetics.
3. Anterior-posterior predominant CTI displacement: when the predominant movement was along the anterior-posterior axis.
4. Caudo-cranial predominant CTI displacement: when the predominant movement was along the caudo-cranial axis.

Segmental analysis revealed a progressive increase in displacement from the EV towards the TV. As shown in *Figure 2C*, the CTI displacement in the anterior-posterior direction increased significantly (*P* < 0.05) from 8.1 ± 7.3 mm at segment 1 (near the EV) to 17.2 ± 8.1 mm at segment 5 (near the TV). Similarly, in the caudo-cranial direction, there was a significant (*P* < 0.05) but less pronounced increase from 1.6 ± 0.8 mm near the EV to 4.5 ± 2.4 mm near the TV. The anterior-posterior displacement was four times greater than the caudo-cranial one. Altogether, segments near the TV exhibited significantly greater displacement compared with those near the EV (15 ± 6 mm vs. 8 ± 6 mm; *P* < 0.05), indicative of a progressive increase in displacement towards the TV.

### Clustering analysis of CTI motion kinetics

**Figure 3:**
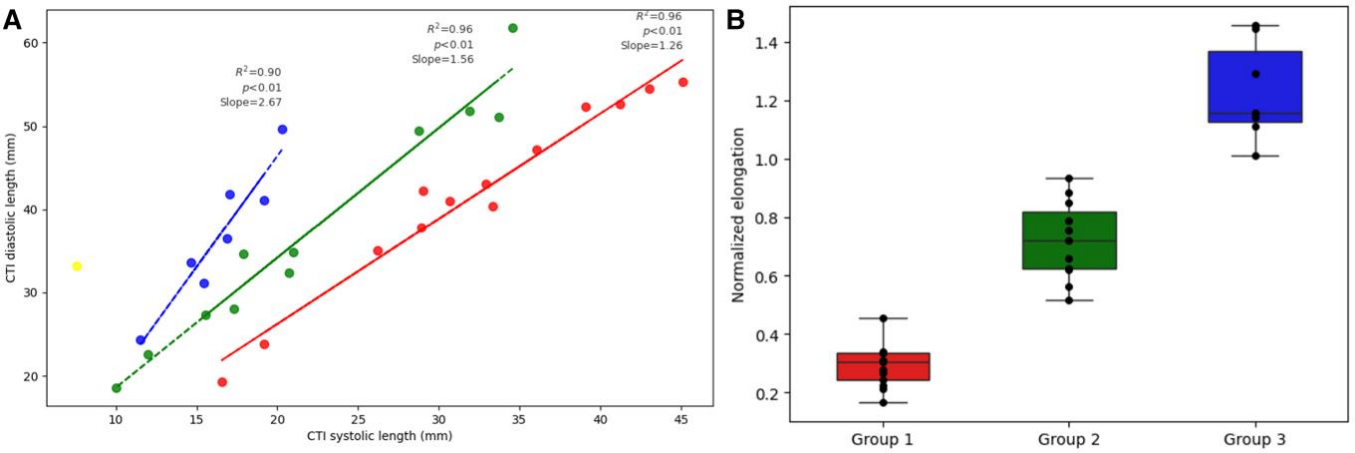
Patients’ clustering based on NE. (*A*) Depiction of NE clusters based on the NE components. (*B*) Depiction of NE value in relationship with the clusters.

*Figure 3A* illustrates the relationship between CTI length in diastole and systole, stratified by NE-based groups. The k-means clustering revealed four distinct groups as shown by the elbow method (see Supplementary data online, *Figure S4*). Upon closer inspection, one of the groups consisted of a single outlier patient (illustrated in yellow), subsequently removed from further analysis.

*Figure 3B* presents the NE for each of the three remaining groups with mean NE values of 0.29, 0.72, and 1.23, referred to as low, intermediate and high NE, respectively, further on. These results show that patients with shorter CTI systolic length are more dynamic, exhibiting higher NE compared with patients with longer CTI systolic lengths. In summary, NE appears to be a more effective measure of CTI dynamicity than the absolute CTI length. This unsupervised classification prompted further investigation into potential associations between NE-based groups and their clinical features.

#### Relationship between CTI metrics and clinical variables

**Figure 4:**
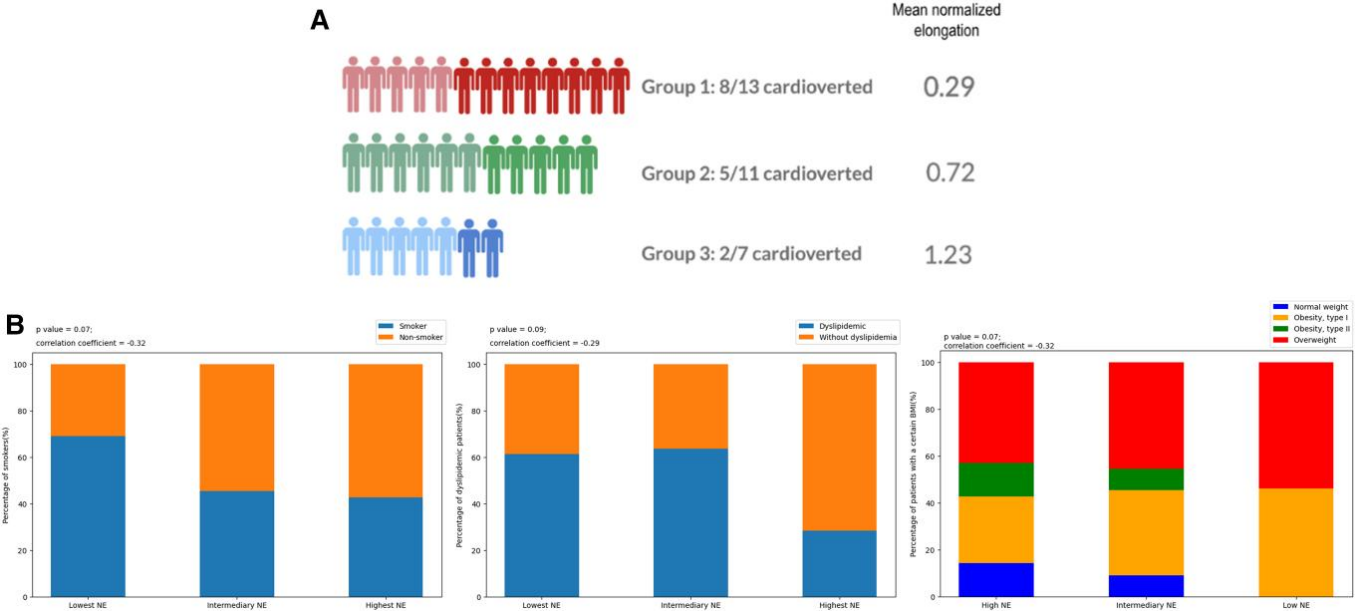
Associations between the NE group and clinical features. (*A*) Relationship between EC and NE. (*B*) NE in relation with comorbidities.

*Figure 4A* illustrates the percentage of patients undergoing EC in each of the three NE-based patient groups. The point bi-serial correlation test for the NE-EC relationship yielded a correlation coefficient of −0.32 (*P* = 0.05), suggesting that patients who were cardioverted within 12 h prior to ablation were more likely to have low NE values. Conversely, patients with a hyperkinetic CTI (i.e. high NE) were less likely to be cardioverted within the same timeframe.

Kolmogorov–Smirnov tests (see Supplementary data online, *Table S3*) revealed significant differences in the distribution of both instantaneous velocity and trajectory differentials for both axes between EC and non-EC patients (*P* < 0.05). EC patients exhibited restricted caudo-cranial (Y-axis) and anterior-posterior (X-axis) movements (i.e. instantaneous velocities on X and Y axis) and displayed lower values than their non-EC counterparts. Importantly, no significant relationship was found between CTI NE values and RF duration (*P* = 0.73; Supplementary data online, *Table S1*).

*Figure 4B* shows that the point bi-serial test for smoking status and dyslipidaemia approached statistical significance with *P*-values of 0.07 and 0.09, respectively. Both variables displayed a negative correlation with NE, with correlation coefficients of −0.32 for smoking and −0.29 for dyslipidaemia. This suggests that smokers and dyslipidaemic patients tend to display low NE. Similarly, there was a borderline correlation (*P* = 0.07) between NE and BMI values (*Figure 4B*, *P*-value of 0.06 and correlation coefficient of −0.32), suggesting that higher BMI tended to be associated with a lower NE (i.e. less dynamic CTI). NYHA class and age were not significantly associated with NE (*P* = 0.36 and *P* = 0.58, respectively), but with the absolute elongation (*r* = −0.38, *P* = 0.03, and *r* = −0.4, *P* = 0.02, respectively). A higher NYHA class or an older patient displayed a reduction in the CTI motion amplitude (see Supplementary data online, *Figure S7*). Absolute elongation also showed a significant correlation with smoking and dyslipidaemia (*r* = −0.35, *P* = 0.05; *r* = −0.32, *P* = 0.04), but no correlation with BMI.

### Impact of clinical and procedural parameters on predicting CTI kinetics and metrics

The accuracy of the logistic regression based on the trajectory differentials was 86.59% on both X and Y axes. For the Euclidean differentials, the accuracy was 87.62%. The confusion matrices are in Table 4. Altogether, the logistic regression was accurate in predicting whether the patient was cardioverted or not based on trajectory differentials vectors.

**Table 4:** Accuracy and confusion matrices for predicting EC based on trajectory differentials vectors

Logistic regression	Accuracy	True negatives	False positives	False negatives	True positives
X-axis	86.59%	30	5	8	54
Y-axis	86.59%	30	4	9	54
2D plane	87.62%	28	7	5	57

**Figure 5:**
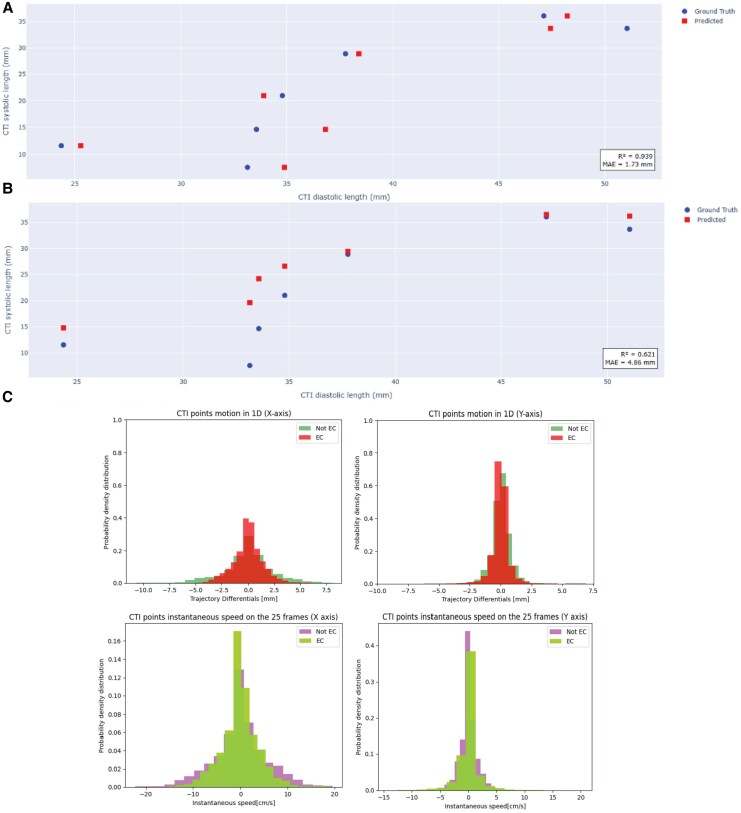
Predictions of CTI systolic and diastolic length using the Lasso regression. (*A*) CTI diastolic length prediction based on curvilinear CTI systolic length, pouch depth, and linear CTI length (concavity parameters). (*B*) CTI systolic length prediction based on curvilinear CTI diastolic length, pouch depth, and linear CTI length (concavity parameters). (*C*) Distribution differences in the trajectory differentials and the instantaneous speeds in the cardioverted versus non-cardioverted individuals.

*Figure 5A* and *B* depict the prediction of CTI diastolic and systolic length respectively. For CTI diastolic length prediction, the mean absolute error (MAE) was 1.73 mm and the *R*^2^ value between the test set and the ground truth was 0.94, respectively. The median CTI diastolic length of the data set was 39.08 mm, with a standard deviation of 11.23 mm. For predicting CTI systolic length, the MAE was 4.86 mm and the *R*^2^ was 0.621. The median CTI systolic length was 20.84 mm with a standard deviation of 10.21 mm. The clinical and procedural parameters (i.e. EC, smoker, and dyslipidaemia status) did not impact the CTI diastolic length prediction based on the Lasso model (similar *R*^2^ and MAE). In contrast, both parameters did impact the CTI systolic length prediction, as the model displayed improved values (*R*^2^ 0.53 vs. 0.621 and MAE 5.63 mm vs. 4.86 mm). The coefficients for the regressors using the six inputs are presented in Table 5.

**Table 5:** Coefficients for each feature of the best Lasso model

	RA systolic/diastolic length	Linear CTI length	Pouch depth	EC	Smoker status	Dyslipidaemia status
Curvilinear CTI diastolic length prediction (alpha = 0.65)	0	1.12	0.57	0	0	0
Curvilinear CTI systolic length prediction (alpha = 0.17)	−0.19	1.06	−0.34	1.77	4.2	0

## Discussion

### Major findings

Our study has four key findings summarized below: (i) CTI segmental analysis performed within an iCMR revealed four motion patterns unrelated to procedural RF duration; (ii) patients referred for AFL ablation can be clustered based on their CTI NE; (iii) NE is significantly and negatively impacted by recent EC, which is suggestive of a stunning effect; and (iv) there is a specific clinical profile of patients with low NE.

### CTI segmental motion patterns

Displacement and velocity of CTI during the cardiac cycle remain poorly known. To our knowledge, this is the first study to provide a detailed analysis of CTI motion in patients referred for ablation. CMR was leveraged to assess the dynamic behaviour of CTI throughout the cardiac cycle. We found considerable variability in motion kinetics across patients, categorized into distinct groups such as hypokinetic or hyperkinetic CTI. The segmental analysis revealed high interpatient variability with several patterns of displacement and velocity, indicative of heterogeneous motion and/or deformation. The CTI segment adjacent to the IVC remained relatively static serving as an anchoring point within the thorax, while the portion near the TV was stretched forward during ventricular contraction. This mechanical heterogeneity along the CTI length is suggestive of non-uniform elasticity of RA tissue and/or variation in electrical activation and mechanical response. This variability in motion kinetics could account for the challenges encountered in achieving by ablation successful bi-directional CTI block, particularly near the TV. Interestingly, Chikata *et al*.^10^ also noted a diminished contact force and the need for longer RF applications for CTI segments near the TV. One of our working hypotheses was that high CTI kinetics would result in prolonged ablation time. Although we could not provide a definitive answer, a trend for longer ablation time was seen for CTI with high NE values (see Supplementary data online, *Table S1*). Whether CTI segments near the TV result in poorer catheter contact and ablation efficacy needs further investigation.

### Clustering of patients with AFL based on CTI NE

The NE-based clustering analysis identified three distinct groups within our population. Direct comparison with existing literature remains challenging as this is the first study to explore NE for the analysis of CTI kinetics. Of note, our unsupervised ML algorithm uncovered new patterns and criteria that may not align easily with conventional clinical standards or established knowledge, given the novelty of performing ablation within an iCMR.

### Correlation and causality between NE and EC

**Figure 6:**
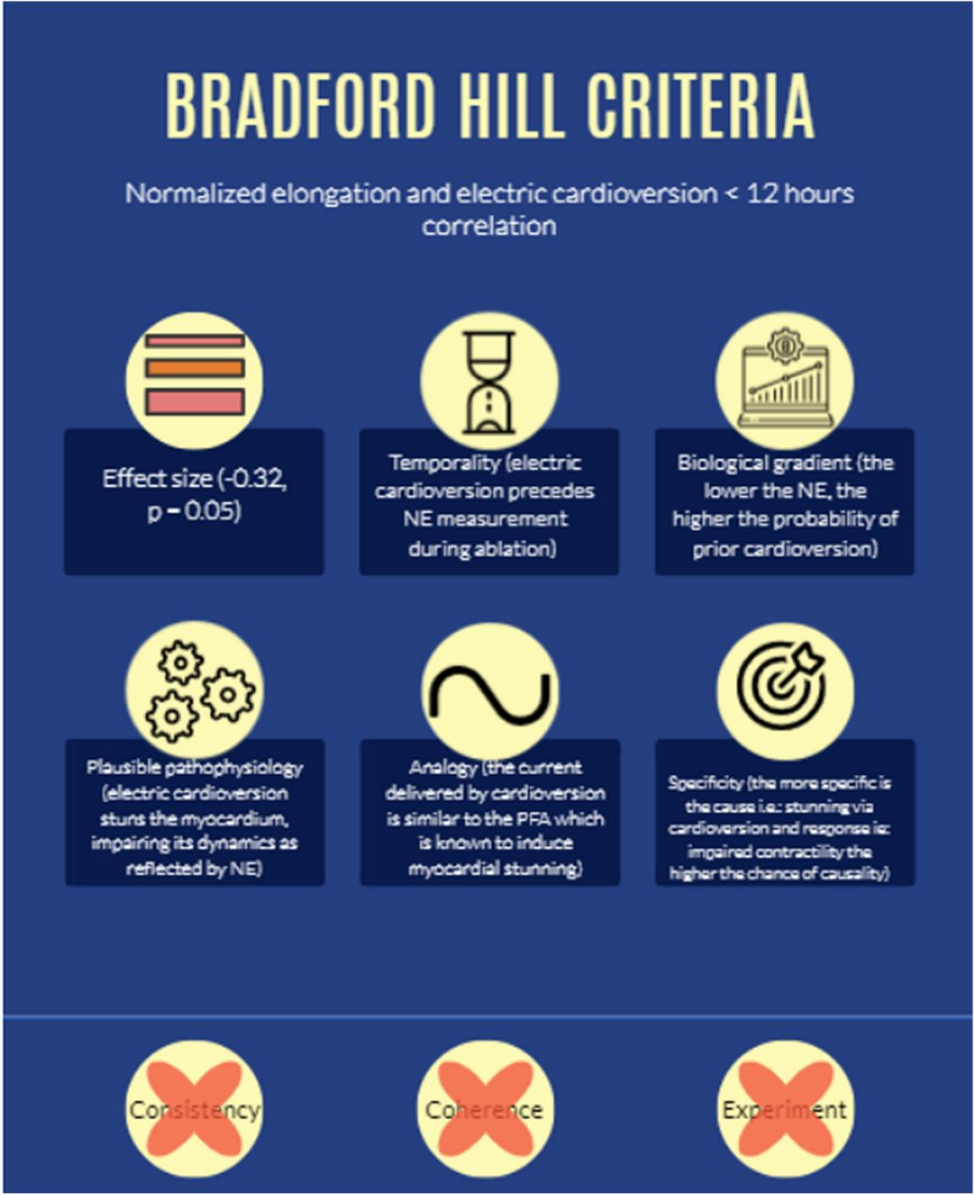
Causality relationship criteria (Bradford Hill) for NE and EC.

The three patient groups identified based on NE values showed a significant correlation with EC performed within 12 h prior to ablation. Specifically, patients who were EC showed lower NE than those who were not. Six out of nine Bradford Hill criteria were fulfilled, suggesting that the relationship between EC and NE may be both associative and causal (*Figure 6*).

1. Effect size: we observed a significant effect size of −0.32 (*P*-value = 0.05).
2. Temporality: all EC preceded NE measurements.
3. Biological gradient: a lower NE was associated with a higher likelihood of prior EC, consistent with a dose-response relationship.
4. Plausibility: the underlying pathophysiological mechanism might involve myocardial stunning, which decreases transiently contractility.^11^
5. Analogy: EC has an effect similar to the electroporation effect seen using pulsed-field ablation, which can induce myocardial stunning.^12^
6. Specificity: the specificity of the cause (EC-induced stunning) and the response (reduced NE) strengthens the case for a causal relationship.

However, criteria of consistency, coherence, and experiment remain to be demonstrated through reproducibility and further translational research (*Figure 6*).

### Clinical profile of patients with low NE

Smoking, which is proinflammatory,^13,14^ has been identified as a factor impairing myocardial contractility^15^ and leading to increased left and right ventricular end-systolic volumes.^16^ Although previous studies have focused on ventricular function,^11^ our study is the first to suggest an association between smoking status and impaired RA contractility as reflected by the reduced NE. The correlations between dyslipidaemia, obesity and NE suggest that patients with low NE tend to have more comorbidities, which might contribute to reduced CTI kinetics. Whether interventions such as dietary restrictions, GLP1 prescription or dyslipidaemia treatment could improve CTI kinetics require further investigation beyond our study. In an unselected population, large RA can be seen in high BMI, which was not found in our patients. Our population had an RA volume like the mean value of the study population of Keller *et al*. (25.8 mL vs. 26.1 mL).^16^

Age and NYHA class are contributing factors to reduced CTI kinetics. Lower absolute elongation values, but not NE, were observed in older individuals or higher NYHA class. Several factors could explain these contrasting findings. Aging is associated with increased myocardial interstitial fibrosis,^17^ which leads to stiffer atrial tissues with reduced compliance, potentially impacting regions like the CTI. Unlike absolute elongation, NE normalizes the elongation by accounting for the initial systolic length of the CTI, providing a relative measure of tissue compliance. We hypothesized that NE characterizes the stiffness or compliance of the CTI, akin to the stiffness constant *k* of a spring in a mechanical system. This analogy helps explain the linear elastic behaviour observed in NE for standard CTI lengths, comparable to a spring operating within the region where the stress and strain are directly proportional. In summary, our findings suggest that low NE values are indicative of reduced compliance reflecting stiffer tissue, while high NE values are suggestive of increased CTI compliance or elasticity and, by extension, of the RA. This approach provides a useful framework to understand the elastic behaviour of the CTI in physiological and pathological conditions.

As shown in Supplementary data online, *Figure S6*, we observed a stronger impact of EC on CTI kinetics along the anterior–posterior axis than along the caudo-cranial one. Non-EC individuals displayed higher instantaneous velocity and greater range of velocity and trajectory differentials. These findings suggest that EC *per se* transiently reduces CTI dynamicity, potentially aligning with a shift towards stiffer, less compliant tissue behaviour.

### Clinical and procedural parameters as contributors to CTI systolic length and kinetics prediction

The Lasso algorithm showed that EC and smoker status played a significant role in predicting the CTI systolic length. Both were among the top three parameters ranked by the Lasso regression coefficients. Interestingly, Leung *et al*. showed that the end-systolic circumferential strain of the left ventricle can be used as a predictor of functional recovery following STEMI.^18^ As the CTI systolic length is part of the NE and as NE is a measure of linear stress-strain regimen, its measurement following EC could serve as a predictor of right atrial functional recovery.

Patients who had low CTI kinetics had a higher likelihood to be predicted as cardioverted. This finding suggests an electroporation-like effect of EC on the CTI, in a similar way that van Zyl *et al*. reported on an animal model.^19^ Also, Fedorov *et al*. showed that the atria are more susceptible to the stunning effect following electroporation.^12^

### Study limitations

The main limitation of this study is its small sample size, which limits our ability to precisely determine correlations between NE and clinical variables or EC, as well as the generalizability of the proposed prediction algorithms. Although the monotonic correlation between NE and ablation duration was not significant (*P* = 0.73), this hypothesis cannot be definitively ruled out due to the limited sample size. Also, our study was unable to satisfy all Bradford–Hill criteria, largely due to the scarcity of literature on NE in ablated animal models and humans. Another limitation is the presence of other potential confounders for the NE-EC causal relationship. The presence of AFL in cardioverted patients might also have affected NE values by decreasing RA contractility. Further studies will shed light on whether AFL or RA contractile remodelling are confounders for the EC-NE relationship. Finally, while the prediction algorithm provided valuable insights, it requires validation to confirm its findings.

## Conclusion

CTI displayed a gradual kinetic pattern along its length, with segments close to the TV undergoing larger displacements than those near the EV. NE, defined as the ratio between the difference in diastolic and systolic CTI lengths over the systolic CTI length, is a useful descriptive kinetic parameter. The clustering algorithm identified three patient groups with low, intermediate, and high NE values. Patients who underwent EC within 12 h prior to the iCMR ablation were more likely to display low NE, but also to be smokers, dyslipidaemic, and obese. The Lasso model highlighted the impact EC and smoker status has on the CTI systolic length prediction, while the logistic regressor pointed out that the CTI kinetics are predictive of EC/non-EC status. NE might become a biomechanical marker of tissue compliance for the linear stress-strain regime of the heart and for right atrial pathology.

## Supplementary Material

qyaf106_Supplementary_Data

## Data Availability

The data underlying this article cannot be shared publicly due to ethical reasons. The data will be shared on request to the corresponding author with permission of the Lausanne University Hospital.

## Lead Author Biography

Dr Alex Mircea graduated yearly in the top 5% from Carol Davila University with a merit scholarship. He completed clinical training in cardiology at Cochin Hospital, Paris, and conducted research on ventricular tachycardias at UCLA. He later rotated at HUG, focusing on Holter data, and trained at Stanford, developing a deep learning model for cancer detection on ultrasound. He earned a master's in electromechanical heart diseases from IHU Lyric. Currently, he is pursuing a PhD jointly at CHUV and Northwestern, with clinical work in cardiac prevention and electrophysiology, and research spanning biomedical engineering and cardiac arrhythmia management.
