# Supplementary material for: Correlation of cavotricuspid isthmus dynamics with clinical parameters: insights from interventional cardiac magnetic resonance imaging
Source: Eur Heart J Imaging Methods Pract. 2025 Oct 8;3(4):qyaf106. doi: 10.1093/ehjimp/qyaf106 (PMC12504068; doi:10.1093/ehjimp/qyaf106)
Supplement: qyaf106_Supplementary_Data [file qyaf106_supplementary_data.docx]

# Supplemental materials

## Supplementary methods

### Ablation procedure

In this procedure, cardiovascular magnetic resonance (CMR)-guided electrophysiological ablation was performed for typical right atrial flutter using a specialized MR-conditional catheter, the Vision-MR from Imricor (Burnsville, MN, USA), alongside an MR-conditional electrophysiological recording system (Advantage-MR, Imricor) and a standard RF-pulse generator (IBI-1500T11, St. Jude Medical, St. Paul, MN, USA). The catheter was specifically designed to reduce MR-induced heating, electrical noise, and imaging artifacts. It had an 8.5 Fr shaft, a 115 cm insertable length, and a deflectable length of 104 mm. Its distal end featured two gold electrodes for high-fidelity electrogram recording and pacing, and a 3.5 mm tip with six irrigation ports for cooling during RF-ablation. The procedure was conducted on a Philips Ingenia 1.5 T MR scanner equipped with Omega HP gradients (45 mT/m, 200 T/m/s) and a 28-element array coil with fully digitized signal transmission. Image guidance was facilitated by an Interventional MR Suite (iSuite) platform, which was fully integrated into the electrophysiological recording system. Venous femoral access (10 Fr sheath) from both sides and arterial access (4 Fr) were obtained outside the scanner room, with sedation administered before transferring the patient to the CMR suite. There was no steerable sheath, nor an intracardiac echography used during the procedure. 3D mapping was part of the process. Current contact force catheters are incompatible with the CMR yet.


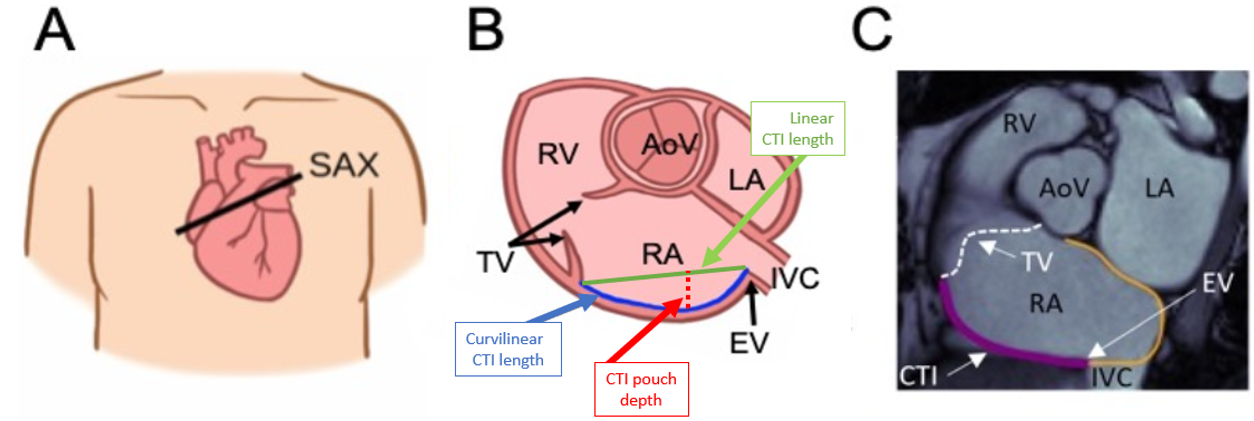


**Supplementary Figure 1.** Schematic view of the heart’s short axis (A) with a tilt and the obtained schematic view (B); and CMR image (C). Panel B shows the two methodologies used for measuring the CTI length, curvilinear and linear.

### Data post-processing

TOMTEC automatically placed 49 points along the previously delineated RA contour (Supplementary Figure 2). This number arises from the inclusion of a critical point located near the IVC, splitting each side into 24 symmetrical points. A variable number pertains to the CTI based on the patient’s cardiac anatomy (e.g. 8-19 points).


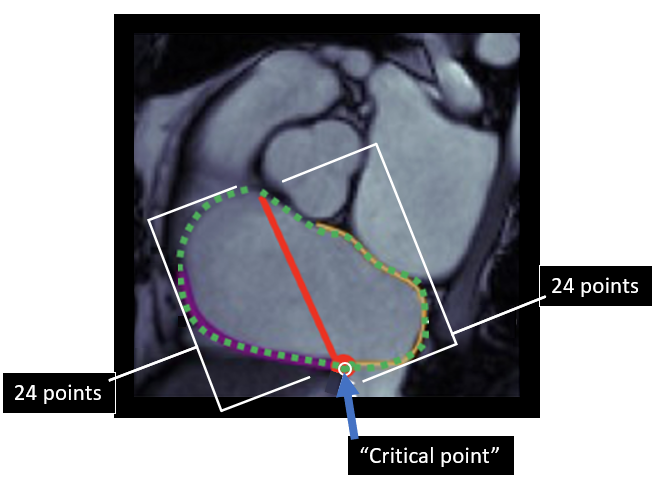


**Supplementary Figure 2.** Right atrium contouring process.

## Supplementary Results

Supplementary Figure 3 displays the trajectories of the middle point of each of the 5 CTI segments during the cardiac cycle from 6 patients.

Supplementary Figure 4 highlights the elbow method used for patients clustering based on normalized elongation. This consists of choosing the number of clusters that reduce the distances between points pertaining to a cluster, while maximizing the distances between clusters

Supplementary Figure 5 underscores the absence of relationships between procedural time and NE/absolute elongation.

Supplementary Figure 6 shows the relationships between normalized elongation, electrical cardioversion and procedural time.

Supplementary Figure 7 shows the relationship between elongation and age/NYHA.

Supplementary Table 1 shows the trend between the NE clusters and the procedural time.

Supplementary Table 2 shows the independence of electrical cardioversion from the clinical variables.

Supplementary Table 3 displays the Kolmogorov-Smirnov test results. Electrically cardioverted patients have narrower ranges of both space differentials and instantaneous speeds.


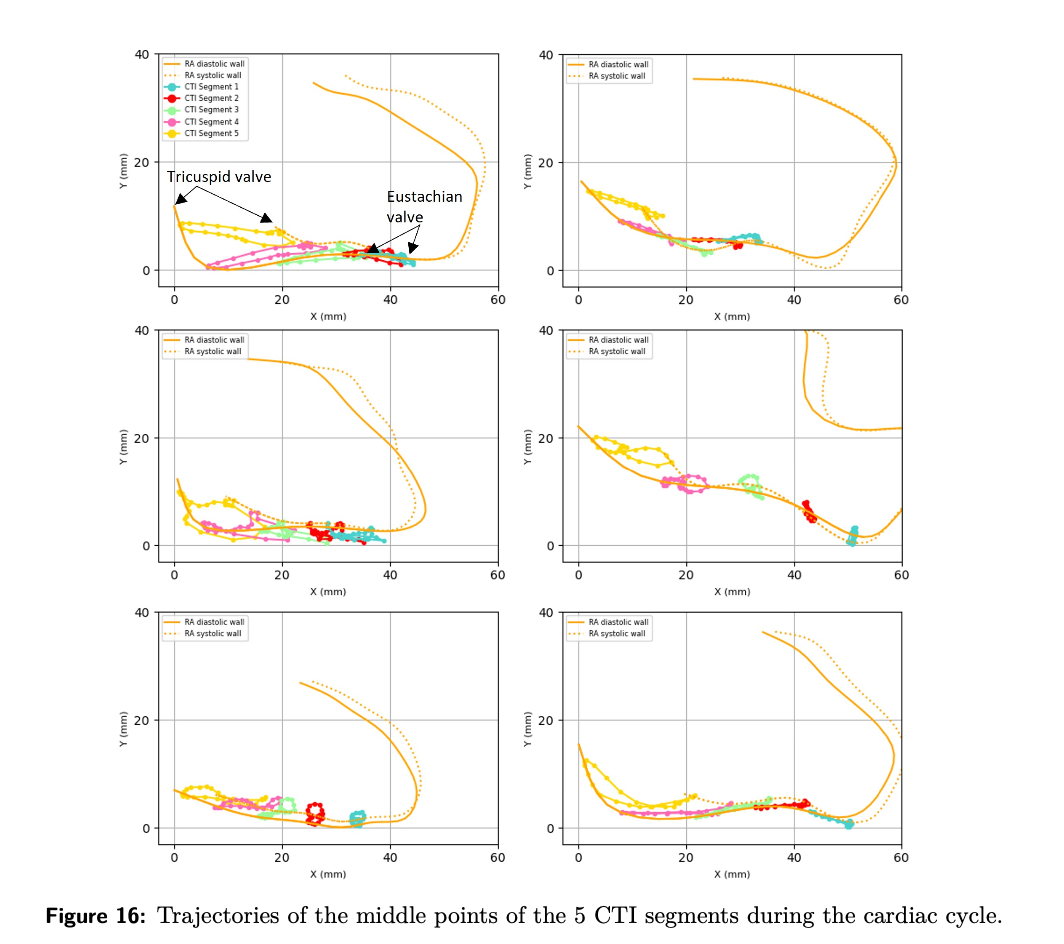


**Supplementary Figure 3.** Representative trajectories of the middle points of each 5 CTI segments during the cardiac cycle from 6 patients


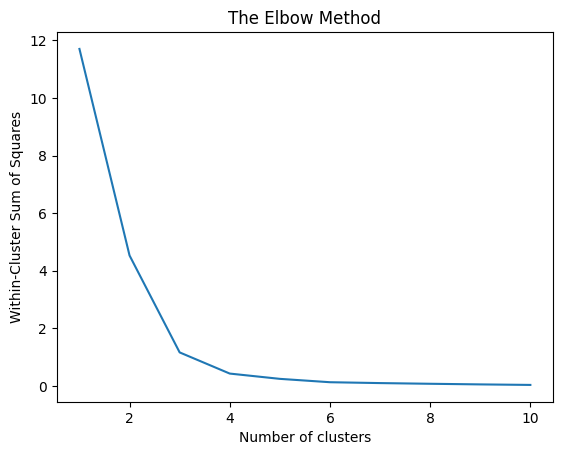


**Supplementary Figure 4.** The elbow method for patients clustering based on normalized elongation.


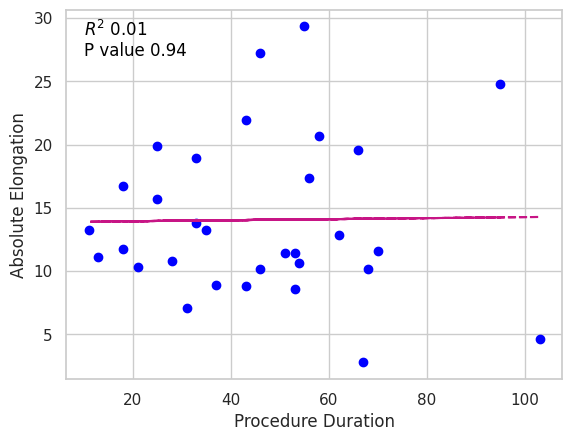

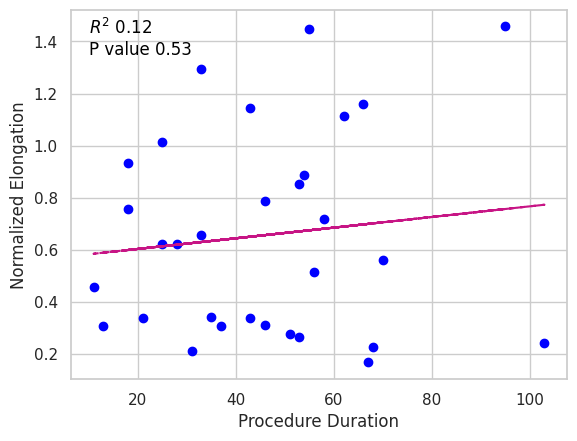


**Supplementary Figure 5.** Absolute and normalized elongation relationship with procedure duration.


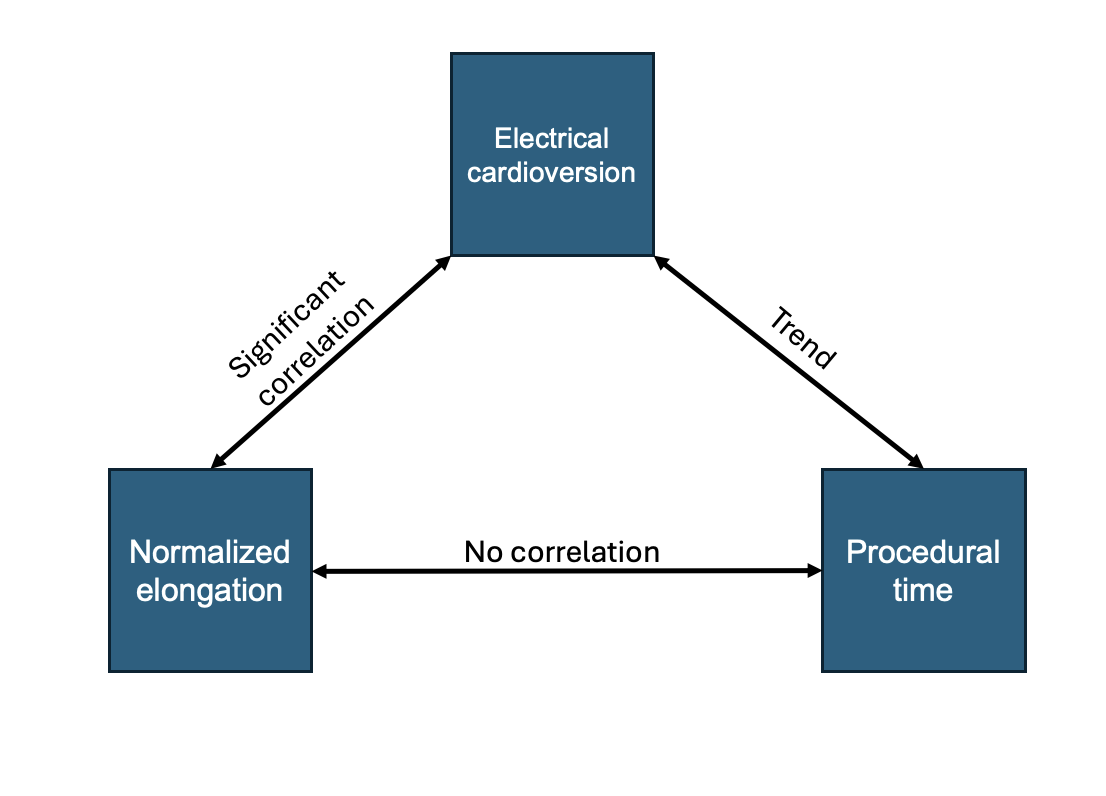


**Supplementary Figure 6.** Relationships between normalized elongation, electrical cardioversion and procedural time.


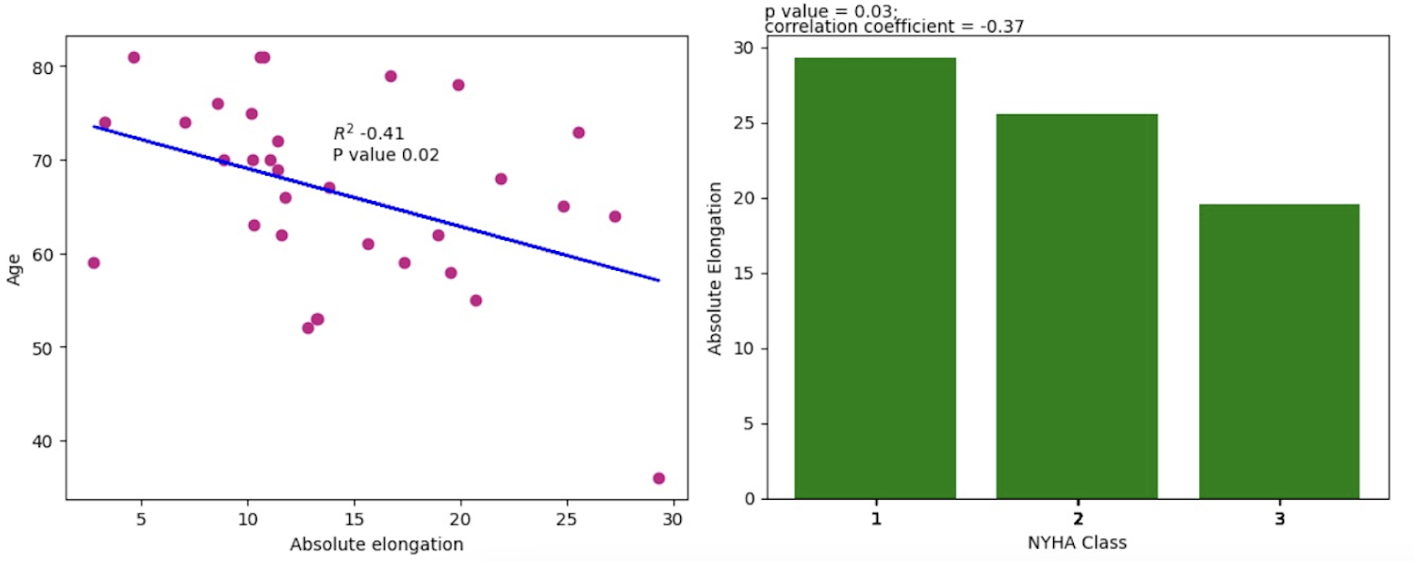


**Supplementary Figure 7.** Relationship between absolute CTI elongation and age/NYHA.

| Group | Mean procedural time |
| --- | --- |
| Low NE | 43 |
| Intermediate NE | 46 |
| High NE | 55 |

**Supplementary Table 1.** Relationship between NE-based clusters and duration of ablation (p=0.73, biserial-point test).

| Relationship | P-Value |
| --- | --- |
| Electrical cardioversion - BMI | 0.9 |
| Electrical cardioversion - Smoker status | 0.48 |
| Electrical cardioversion - Dyslipidemia | 0.48 |

**Supplementary Table 2.** Independence of electrical cardioversion and clinical variables.


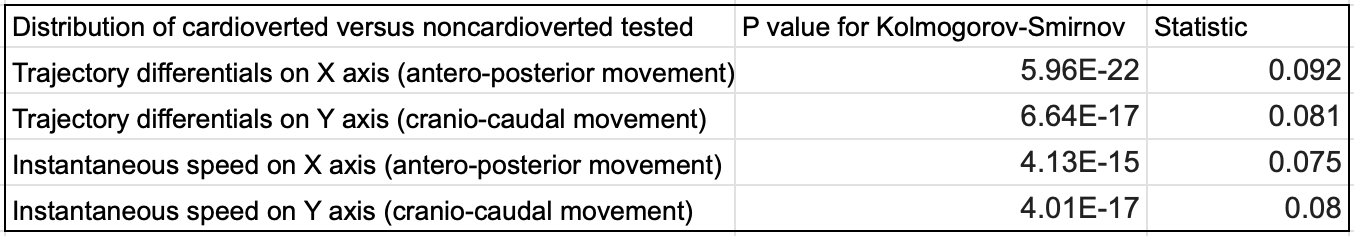


**Supplementary Table 3.** Distribution of trajectory differentials and instantaneous speeds on X and Y axis & their Kolmogorov-Smirnov tests (cardioverted versus non-cardioverted).
